# Culture of human alveolar epithelial type II cells by sprouting

**DOI:** 10.1186/s12931-018-0906-9

**Published:** 2018-10-19

**Authors:** Petra Khan, Kleanthis Fytianos, Luca Tamò, Michael Roth, Michael Tamm, Thomas Geiser, Amiq Gazdhar, Katrin E Hostettler

**Affiliations:** 1 Department of Biomedicine and Clinics of Respiratory Medicine, University Hospital Basel, University of Basel, Petersgraben 4, 4031 Basel, Switzerland; 2 Department of Pulmonary Medicine, University Hospital, University of Bern, 3010 Bern, Switzerland

**Keywords:** Alveolar epithelium, Cell culture, Primary human cell

## Abstract

**Background:**

Type II alveolar epithelial cells (AT2) play a pivotal role in maintaining the integrity and function of the alveoli. Only recently, the role of impaired epithelial repair mechanisms after injury in the pathogenesis of idiopathic pulmonary fibrosis has been demonstrated, and has shifted the AT2 cell in the focus of interest. Therefore, using primary human AT2 cells instead of cell lines for in vitro experiments has become desirable. Several groups have developed methods to isolate human AT2 cells applying tissue digestion and consecutive filtration in their protocols. Here we present a technique to isolate primary human AT2 cells by sprouting directly from peripheral human lung tissue.

**Methods:**

Epithelial cell cultures were established from lung tissue obtained from patients undergoing diagnostic or therapeutic video-assisted thoracoscopic surgery or undergoing flexible bronchoscopy with transbronchial biopsy.

Lung tissue was cut into small pieces and those were placed into cell culture flasks containing supplemented epithelial growth medium for cell sprouting. Cells were characterized by immunofluorescence stainings for E-cadherin, pan-cytokeratin, surfactant protein C (SP-C), and for lysotracker; fluorescent surfactant associated protein B (SP-B) uptake and secretion was assessed by live cell imaging; RNA levels of SP-A, SP-B, SP-C, and SP-D were determined by real-time PCR; Electron microscopy was used to search for the presence of lamellar bodies.

**Results:**

Sprouting of cells started two to four days after the start of culture. Epithelial differentiation was confirmed by positive staining for E-cadherin and pan-cytokeratin. Further characterization demonstrated positivity for the AT2 cell marker SP-C and for lysotracker which selectively labels lamellar bodies in cultured AT2 cells. The up-take and release of SP-B, a mechanism described for AT2 cells only, was demonstrated by live cell imaging. Real-time RT-PCR showed mRNA expression of all four surfactant proteins with highest levels for SP-B. The presence of lamellar bodies was demonstrated by electron microscopy.

**Conclusions:**

This study describes a novel method for isolating AT2 cells from human adult lung tissue by sprouting. The characterization of the cultured AT2 cells complies with current criteria for an alveolar type 2 cell phenotype. Compared to current protocols for the culture of AT2 cells, isolating the cells by sprouting is simple, avoids proteolytic tissue digestion, and has the advantage to be successful even from as few tissue as attained from a transbronchial forceps biopsy.

## To the editor

The lung alveolar epithelium comprises two types of specialized epithelial cells, the alveolar epithelial type I cells (AT1), that cover approximately 93% of the alveolar surface area and through which gas exchange takes place, and the type II alveolar epithelial cells (AT2) that constitute 60% of lung alveolar cells and are the producer of the different surfactant proteins [1–3]. AT2 cells play a pivotal role in maintaining the integrity and function of the alveoli and serve as progenitor of AT1 cells [4–6]. Lung injury by agents such as cigarette smoke, viruses, and environmental particles mainly target the alveolar epithelium [7], emphasizing its role for tissue homeostasis [5]. Only recently, the role of impaired repair mechanisms after injury in the pathogenesis of idiopathic pulmonary fibrosis has been demonstrated [8, 9], and has shifted the AT2 cell in the focus of interest. Therefore, using primary human AT2 cells instead of cell lines (e.g. A549 epithelial cells) for in vitro experiments has become desirable. Several groups have developed methods to isolate human AT2 cells, all applying tissue digestion (using trypsin or elastase) and consecutive filtration in their protocols [10–13]. Here we present a technique to isolate primary human AT2 cells by sprouting directly from peripheral human lung tissue.

## Materials and methods

### Ethical approval

Human lung tissue was obtained with approval of the Human Ethics Committee of the University of Basel (EKBB 05/06) and written informed consent was obtained from all patients who underwent lung biopsy.

### Patients

Epithelial cell cultures were established from lung tissue obtained from patients undergoing diagnostic or therapeutic video-assisted thoracoscopic surgery (VATS) performed at the Division of Thoracic Surgery or undergoing flexible bronchoscopy with transbronchial biopsy at the Clinics of Respiratory Medicine, University Hospital Basel, Switzerland. In patients with lung tumors, lung tissue for cell culture was obtained from the macroscopically normal part away from the tumor.

### Cell culture

Lung tissue was cut into small pieces and those were placed into cell culture flasks for cell sprouting containing supplemented epithelial growth medium (Cnt-17) (CELLnTEC Advanced Cell System AB; Bern, Switzerland). AT2 cells were grown under standard conditions (37 °C, 21% O_2_, 5% CO_2_). Complete epithelial culture medium was replaced every fourth day. All experiments were performed using non-passaged cells. Primary human lung fibroblasts cultured in DMEM/10% FCS were used as negative controls [14].

### Immunofluorescence stainings in cultured cells

Immunofluorescence analysis of AT2 cells or fibroblasts was performed as previously described [15]: cells were fixed with 4% formalin (10 min), and permeabilized with methanol/acetic acid (3:1, ice-cold, 10 min). Cells were blocked with 5% BSA (1 h), and then incubated with TRIC-Phalloidin (Sigma) for 1 h at room temperature or antibodies against E-cadherin (Abcam, Santa Cruz, LabForce AG), pan-cytokeratin (Sigma-Aldrich, USA), calponin (Abcam), surfactant protein C (SP-C) (ThermoFisher Scientific), overnight at 4 °C in moist box; for LysoTracker® Green DND-26 (Cell Signaling Technologies, USA) cells were incubated for 30 min at 37 degree. The primary antibodies were detected by addition of Alexa Fluor 488 donkey anti-rabbit, Alexa Fluor 594 goat anti-rabbit or Alexa Fluor donkey anti-mouse IgG (all ThermoFisher Scientific) for 1 h. To visualise the nuclei 4,6-diamido-2-phenylindole (Sigma) was added (5 min) and cells were subsequently examined on a fluorescence microscope Leica DMI4000B (Leica, Germany).

### Live cell imaging

For live cell imaging of fluorescent surfactant associated protein B (SP-B)uptake and secretion, 10 μg of recombinant SP-B (Cloud-Clone Corp, USA) was labelled using BODIPY® FL NHS Ester (ThermoFischer, USA) as described [16] and incubated in a 5% CO2 environment. 8 μg/ml of the resulting fluorescent SP-B were added to each well for 30 min and then rinsed with PBS. The first baseline picture was taken. Later, 100 μM of a diacylglycerol analog (DAG; 1-Oleoyl-2-acetyl-sn-glycerol; Sigma Aldrich,USA) was added and pictures were taken after 5, 15 and 30 min using a Leica DMI4000B (Leica, Germany). The fluorescence intensity was quantified using Image J software (NIH, USA), and data is presented as mean fluorescence intensity.

### Real-time RT-PCR

Total RNA was extracted from AT2 cells with a Quick-RNA MiniPrep Kit (ZymoResearch, Orange, CA) from four different epithelial cell lines. RNA levels were determined by real-time PCR using an SP-A (Hs00831305_s1), SP-B (Hs01090667_m1), SP-C (Hs00161628), SP-D (Hs01108490_m1), or GAPDH (Hs03929097_g1) TaqMan® Gene Expression Assay (all Applied Biosystems, Foster City, CA). Samples were run at 50 °C for 2 min, 1 cycle; 95 °C for 10 min, 1 cycle; 95 °C for 15 s, 60 °C for 1 min, 40 cycle and quantified by the ΔΔCt calculation method.

### Electron microscopy

Cells were grown to confluency in 6-well plates and were fixed with 2.5% glutaraldehyde in 0.15 M Hepes buffer (707 mOsm, pH 7.4) and stored in the fixative solution. The cells were further rinsed with the same fixative and postfixed for 1 h with a solution of 0.1 M sodium cacodylate buffer with 1% solution of osmium teratoxide (369 mOsm, pH 7.4). After rinsing with 0.05 M maleate buffer (pH 5.0), cells were again postfixed in a solution of 0.05 M maleate buffer containing 0.5% uranyl acetate. After 1 h of incubation, cells were dehydrated in consequently increasing concentrations of ethanol (70%, 80%, 96%, 100%) and then left in a mixture of ethanol and epon (1:1) overnight. On the following day, cells were embedded in epon resin and left to polymerize at 60 °C for 6 days. Ultrathin sections (70 nm) were cut with a Reichert-Jung Ultracut E microtome, put on formyar coated 2 mm × 1 mm single slot copper grids and double stained with 1% uranyl acetate and 3% lead citrate. EM pictures were taken with the Philips EM 400 transmission electron microscope, using Morada digital camera and iTEM/cell^F program.

### Western blot

Alveolar epithelial cells from two different donors were used (i.e. sample-1 and sample-2). The cells were lysed with Pierce IP lysis buffer (Thermo Fischer Scientific, Waltham, MA, USA) according to the manufacturer’s specifications. Protein content was analyzed with Bradford assay (BIO-RAD, Hercules, CA, USA) as described in the manufacturer’s protocol. 10 μL of loading buffer (LI-COR, Lincoln, NE, USA) containing 10% β-Mercapto-ethanol (Sigma-Aldrich, St Louis, MI, USA) were added to the samples and incubation at 95 °C for 5 min followed. Samples were shortly centrifuged and were loaded on 12% gels (BIO-RAD, Hercules, CA, USA). A protein molecular weight marker was added (LI-QOR, Lincoln, NE, USA). Gel electrophoresis was performed for 20 min at 80 V and 1 h at 150 V. Gels were transferred to Nitrocellulose membranes (BIO-RAD) by Trans-Blot Turbo Transfer System (BIO-RAD). B-actin (LI-QOR, Lincoln, NE, USA) was used as a control. SP-C (Santa Cruz Biotechnology, Santa Cruz, CA, USA) and β–Actin were added at 1:1000 dilutions and gels were incubated overnight at 4 °C, protected from light with gentle shaking. Gels were washed and incubated with secondary antibodies for 1 h in the dark at room temperature. Secondary antibodies were purchased from LI-COR (IRDye-680RD and IRDye-800RD) and visualization was done with a LI-COR Odyssey scanner.

### Measurement of secreted phospholipids

AT2 cells were serum deprived for 24 h. The cell supernatants were collected and centrifuged at 1200 rpm for 10 min. Epithelial growth media was used as control. Phospholipid secretion was measured by a Phospholipid Assay Kit (Sigma, Aldrich USA) according to the manufacturer’s instructions and data were expressed as concentration of phospholipids in μM.

## Results

**Fig. 1:**
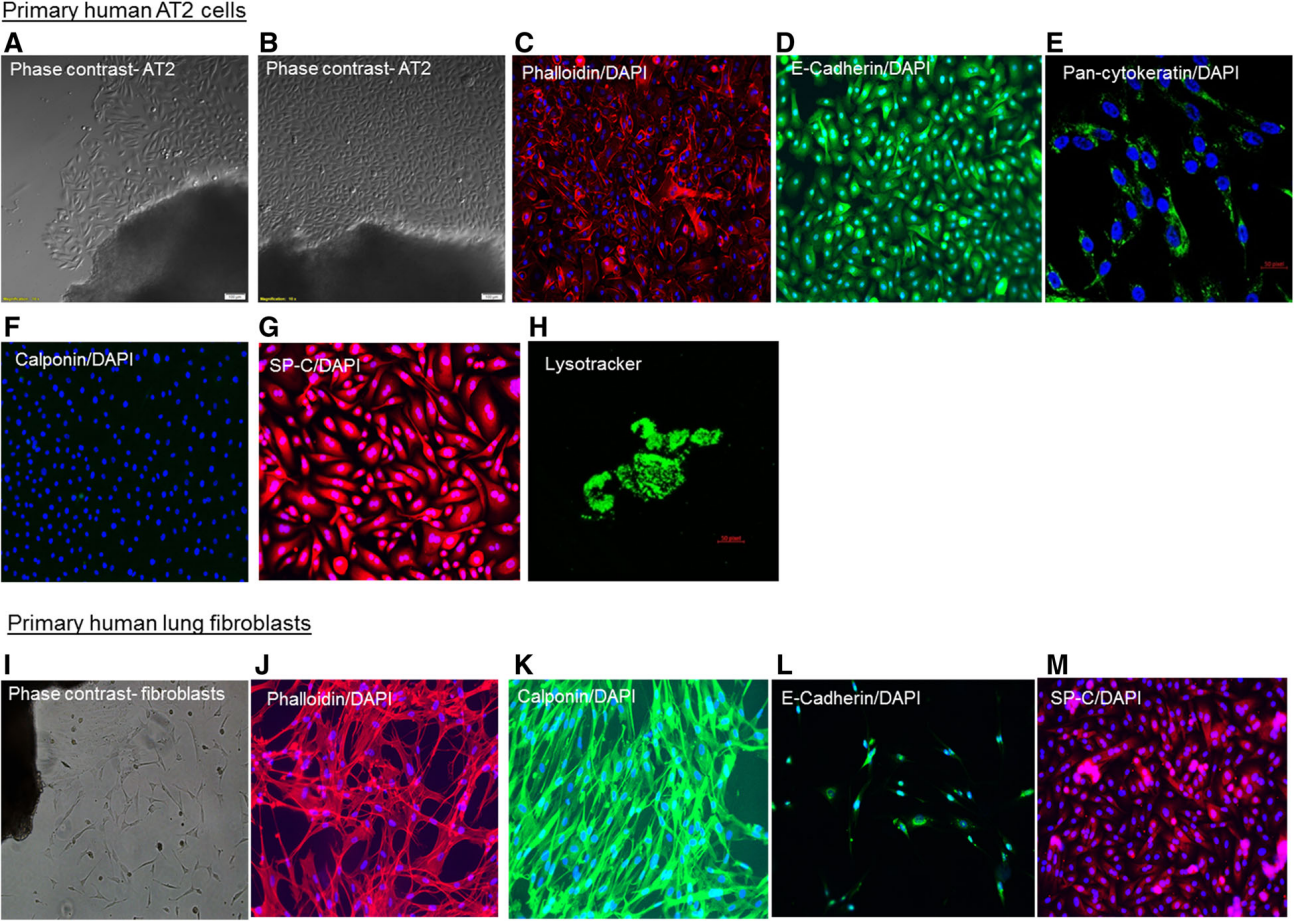
Representative phase contrast pictures of primary human type II alveolar epithelial (AT2) cells (**a**, **b**) and primary human lung fibroblasts (**i**). Phalloidin stainings visualizing the actin cytoskeleton of AT2 (**c**) and fibroblasts (**j**). Immunostainings for E-cadherin (**d**, **l**), pan-cytokeratin (**e**), calponin (**f**, **k**), surfactant protein C (**g**, **m**), and for lysotracker (**h**)

Sprouting of cells started two to four days after the start of culture (Fig. 1a). Cells grew in a monolayer exhibiting a typical cobblestone morphology (Fig. 1b and c). Epithelial differentiation was confirmed by positive staining for the epithelial markers E-cadherin and pan-cytokeratin (Fig. 1d and e) and the absence of the mesenchymal cell marker calponin (Fig. 1f). Further characterization by immunofluorescence stainings demonstrated positivity for the AT2 cell marker SP-C (Fig. 1g) and for lysotracker which selectively labels lamellar bodies in cultured AT2 cells [17, 18] (Fig. 1h). Outgrowth of fibroblasts is observed in very rare cases and can be easily detected by the typical spindle shaped morphology of fibroblasts (Fig. 1i and j), the presence of the mesenchymal marker calponin (Fig. 1k), and the absence of the epithelial marker E-Cadherin (Fig. 1l) and AT2 cell marker SP-C (Fig. 1m).

**Fig. 2:**
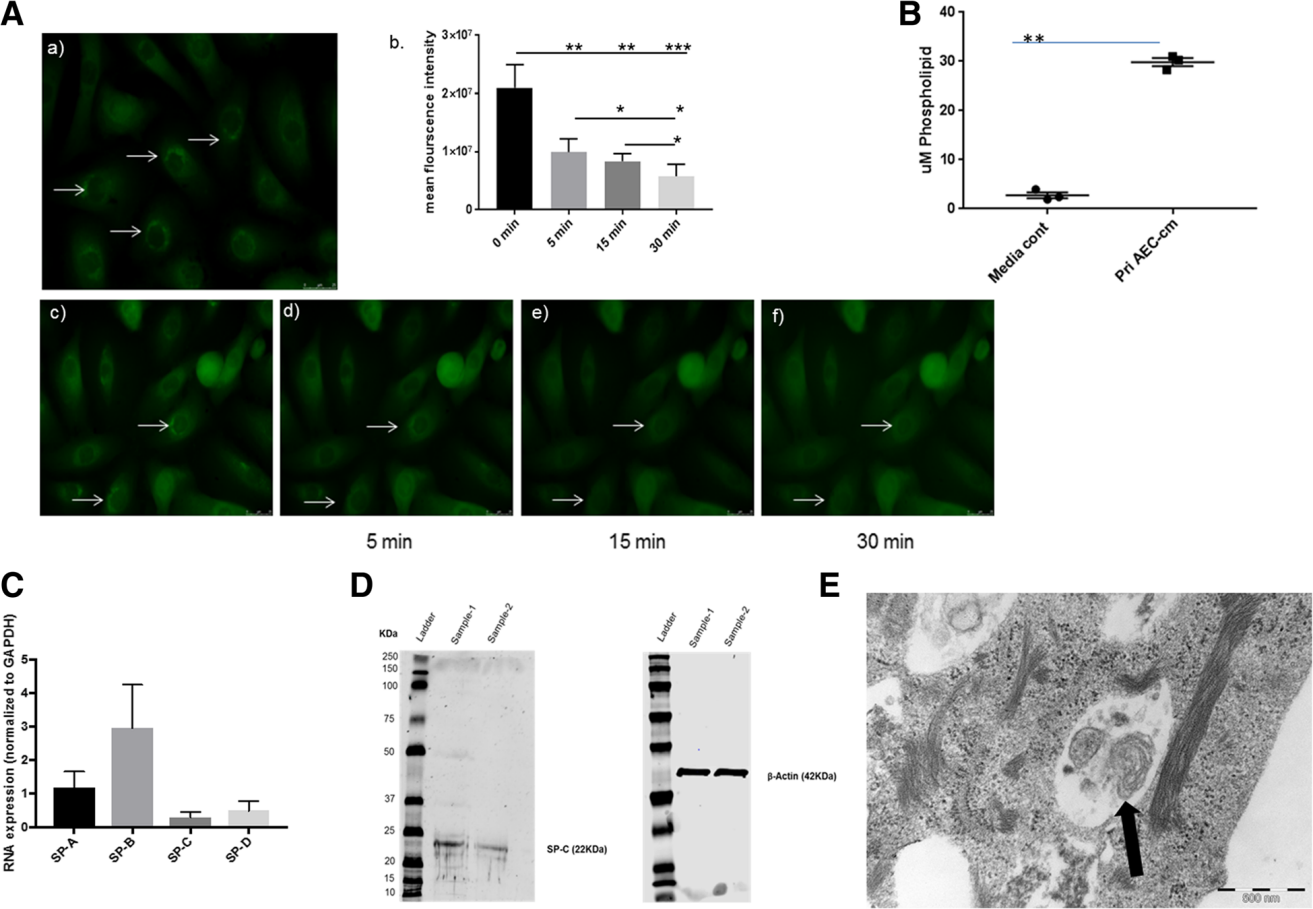
(**a**) Up-take and secretion of labelled surfactant protein B (SP-B) by primary human type II alveolar epithelial (AT2) cells. Labelled SP-B was added to the cells and a first uptake was observed at 0 min (**a** *a*), visualized as green staining in the cytoplasm. Five minutes after addition of a surfactant secretagogue (1-oleoyl-2-acetyl-*sn*-glycerol) which stimulates the secretion of SP-B, a decrease in the fluorescence was detected, and after 15 and 30 min the intensity of fluorescence further diminished (**a** *b-f*). (**b**) Levels of phospholipids (μM) measured in AT2 cell supernatants as compared to control medium. (**c**) Quantitative real time RT-PCR for gene expression of surfactant protein A (SP-A), surfactant protein B (SP-B), surfactant protein C (SP-C), and surfactant protein D (SP-D) by AT2 cells. Relative mRNA expression was normalized to GAPDH. Data are presented as mean ± SEM of independent experiments performed in four different cell lines. (**d**) SP-C protein expression in AT2 cells of two different donors (sample 1 + 2) was determined by western blot. β-actin served as control for equal protein loading. (**e**) Electron microscopy image of AT2 cells showing lamellar bodies (arrow)

In addition, the up-take and release of SP-B was studied in the cells, a mechanism described for AT2 cells only [19–24]. After incubation with labelled SP-B, a first uptake was observed at 0 min (Fig. 2A, a), visualized as green staining in the cytoplasm. Addition of a surfactant secretagogue (1-oleoyl-2-acetyl-*sn*-glycerol) resulted in a significant and time-dependent secretion of SP-B, which was measured by a decrease in fluorescence intensity (Fig. 2A, b). Five minutes after addition, a significant decrease in the fluorescence was detected, and after 15 and 30 min the intensity of fluorescence further diminished (Fig. 2A, b-f). The secretion of phospholipids, the main component of surfactant, was detected in cell supernatants of AT2 cells (Fig. 2b). Furthermore, gene expression of surfactant proteins A, B, C, and D was studied by real-time RT-PCR, showing mRNA expression of all four proteins with highest levels for SP-B (Fig. 2c). In addition, the protein expression of SP-C was confirmed by western blot analysis (Fig. 2d). Finally, the presence of lamellar bodies, a specific feature of AT2 cells, was demonstrated in the cells using electron microscopy (Fig. 2e).

AT2 cell isolation by sprouting from a 1–2 mm piece of peripheral lung tissue resulted in an average yield of 59.5 × 10^3^ ± 12.0 × 10^3^ AT2 cells/tissue piece (*n* = 4 different lung tissue donors) after 7 days of culture. Potential trans-differentiation into AT1 cells was assessed by repeated SP-C immunofluorescence stainings: AT2 phenotype persisted for at least 7 days after start of culture; After 14 days, intensity of SP-C staining decreased (data not shown).

## Discussion

Here we report a technique to isolate primary human AT2 cells by sprouting directly from peripheral human lung tissue. The cells exhibited the typical cobblestone morphology of alveolar epithelial cells, expressed all four surfactant proteins, had the ability to take up and release SP-B, and showed ultrastructural evidence of lamellar bodies.

Using primary human AT2 cells in biomedical research is of utmost importance due to the lack of reliably representative cell lines. A549 human lung adenocarcinoma epithelial cells are often used as a model for studying the function of AT2 cells. However, whereas the cells exhibit some characteristics of AT2 cells, they do not express surfactant proteins [10, 25] and have different cellular phenotypes as compared to AT2 cells [26].

In contrast to previously published methods for culturing AT2 cells, the here described protocol does without initial tissue digestion by trypsin or elastase [10–13], but works by direct cell sprouting from the edge of the tissue. This might prove an advantage of the here presented method, as the use of proteolytic enzymes may alter certain cellular functions or receptor expressions of isolated cells, as demonstrated by trypsin-induced down-regulation of cell adhesion proteins [27–29]. Furthermore, the here presented method allows the culture of AT2 cells starting with as few tissue (approximately 1 mm^3^) as attained from a transbronchial forceps biopsy. In contrast, culture protocols applying tissue digestion and filtration require/start with tissue quantities between 10 g and whole lung lobes [10, 13, 30, 31]. If studying rare diseases such as idiopathic pulmonary fibrosis, access to lung tissue derived from surgical lung biopsies is scarce, and therefore, establishing AT2 cell cultures from transbronchial biopsies is advantageous.

According to recently proposed criteria for the identification of AT2 cells [32], expression of key markers including SP-A, SP-B, SP-C, SP-D and lysotracker was positive in the above described cells. While mRNA for SP-A, SP-B, and SP-D can be found in non-ciliated bronchiolar cells [33], SP-C is the only AT2 cell–specific protein in the adult [32]. Likewise, the uptake and secretion of SP-B was demonstrated in the here characterized cells which is uniquely observed in AT2 cells [19–24]. Release of moderate amounts of phospholipids from these cells was also observed. Finally, ultrastructural evidence of lamellar bodies implies sufficient expression of surfactant proteins [32]. Here, the presence of lamellar bodies was demonstrated by electron microscopy and by positive stainings for lysotracker, a marker of differentiated AT2 cells which selectively labels lipid rich lamellar bodies [18].

## Conclusion

This study describes a novel method for isolating AT2 cells from human adult lung tissue by sprouting. The characterization of the cultured AT2 cells complies with current criteria for an alveolar type 2 cell phenotype. Compared to current protocols for the culture of AT2 cells, isolating the cells by sprouting is simple and avoids proteolytic tissue digestion.

## Abbreviations

AT1: alveolar epithelial type I cells
AT2: alveolar epithelial type II cells
SP-A: surfactant protein A
SP-B: surfactant protein B
SP-C: surfactant protein C
SP-D: surfactant protein D
VATS: video-assisted thoracoscopic surgery
